# Paradoxical pustular psoriasis induced by tumor necrosis factor inhibitor with elevated interferon-alpha in an ankylosing spondylitis patient: A case report

**DOI:** 10.1016/j.jdcr.2025.05.012

**Published:** 2025-06-09

**Authors:** Tinh Khampaen, Monthanat Ploydaeng

**Affiliations:** a Division of Allergy, Immunology, and Rheumatology, Department of Medicine, Faculty of Medicine Ramathibodi Hospital, Mahidol University, Bangkok, Thailand; b Division of Dermatology, Department of Medicine, Faculty of Medicine Ramathibodi Hospital, Mahidol University, Bangkok, Thailand

**Keywords:** ankylosing spondylitis, biologics, cytokine, drug, IFN-alpha, psoriasis, TNF inhibitor

## Introduction

Spondyloarthropathies, particularly ankylosing spondylitis, are inflammatory rheumatic diseases that can cause chronic pain and disability. When patients do not respond adequately to nonsteroidal anti-inflammatory drugs, biologics such as tumor necrosis factor-alpha (TNF-α) inhibitors—including golimumab, adalimumab, and infliximab—are commonly prescribed.^1^ These agents have been shown to significantly improve symptoms and enhance the quality of life for patients.

However, despite their efficacy, clinicians must remain vigilant for paradoxical reactions, such as the development or worsening of psoriasis, reported in some patients undergoing TNF inhibitor therapy. The mechanisms underlying this paradoxical psoriasis are not yet well understood. Some studies suggest that dysregulation of interferon-alpha (IFN-α) levels by uncontrolled plasmacytoid dendritic cells (pDCs) may play a critical role,^2^^,^^3^ while other theories implicate the IL-17/IL-23 axis.^3^ Nonetheless, there is insufficient empirical data directly linking these cytokine changes to TNF-α inhibitor-induced psoriasis, hindering the establishment of a definitive causal relationship.

## Case presentation

A 58-year-old male with a 5-year history of ankylosing spondylitis presented with a rash that developed 2 weeks prior. His medical history included recurrent anterior uveitis treated with azathioprine at 50 mg per day. Due to inadequate responses to nonsteroidal anti-inflammatory drugs and azathioprine, he initiated golimumab therapy at 50 mg monthly 3 years ago, later reducing the dosage to 50 mg every 2 months after 2 years of significant improvement.

The patient had no previous diagnoses of psoriasis or significant skin conditions and reported no new medications or lifestyle changes prior to the rash. His family history included rheumatoid arthritis, and he denied recent infections or stressors.

**Fig 1:**
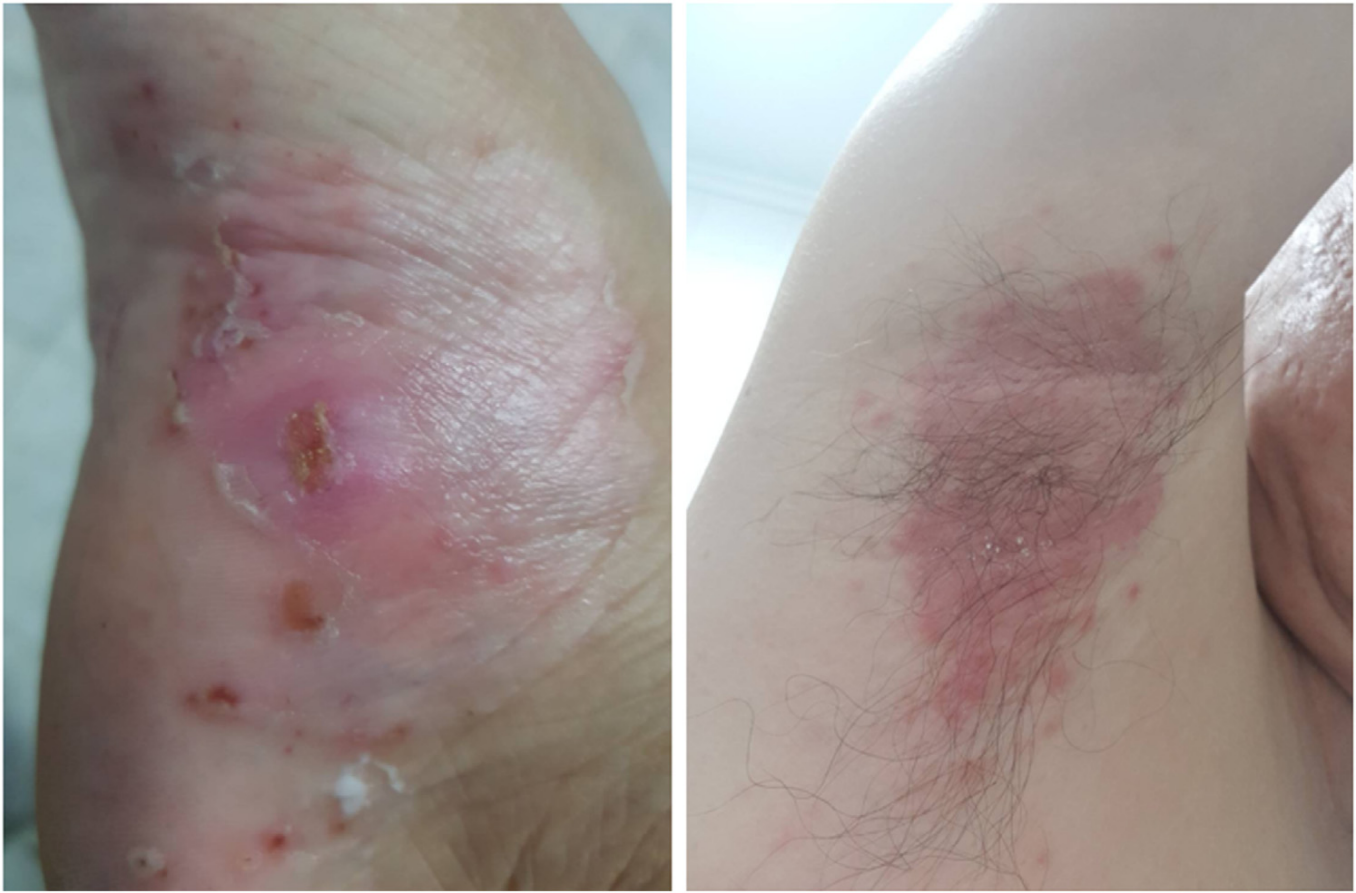
Rashes located on the patient's sole (*left*) and axilla (*right*).

Examination revealed multiple well-defined erythematous plaques with superficial pustules and yellowish crusts, primarily on the palms and soles, with some involvement of the axillary region (Fig 1). The specific distribution of the rash, particularly on the palms and soles, is characteristic of pustular psoriasis, leading to a suspicion of TNF inhibitor-induced psoriasis. Other potential diagnoses included cutaneous candidiasis and irritant or allergic contact dermatitis, although these were less likely given the absence of new medications or treatment changes.

**Fig 2:**
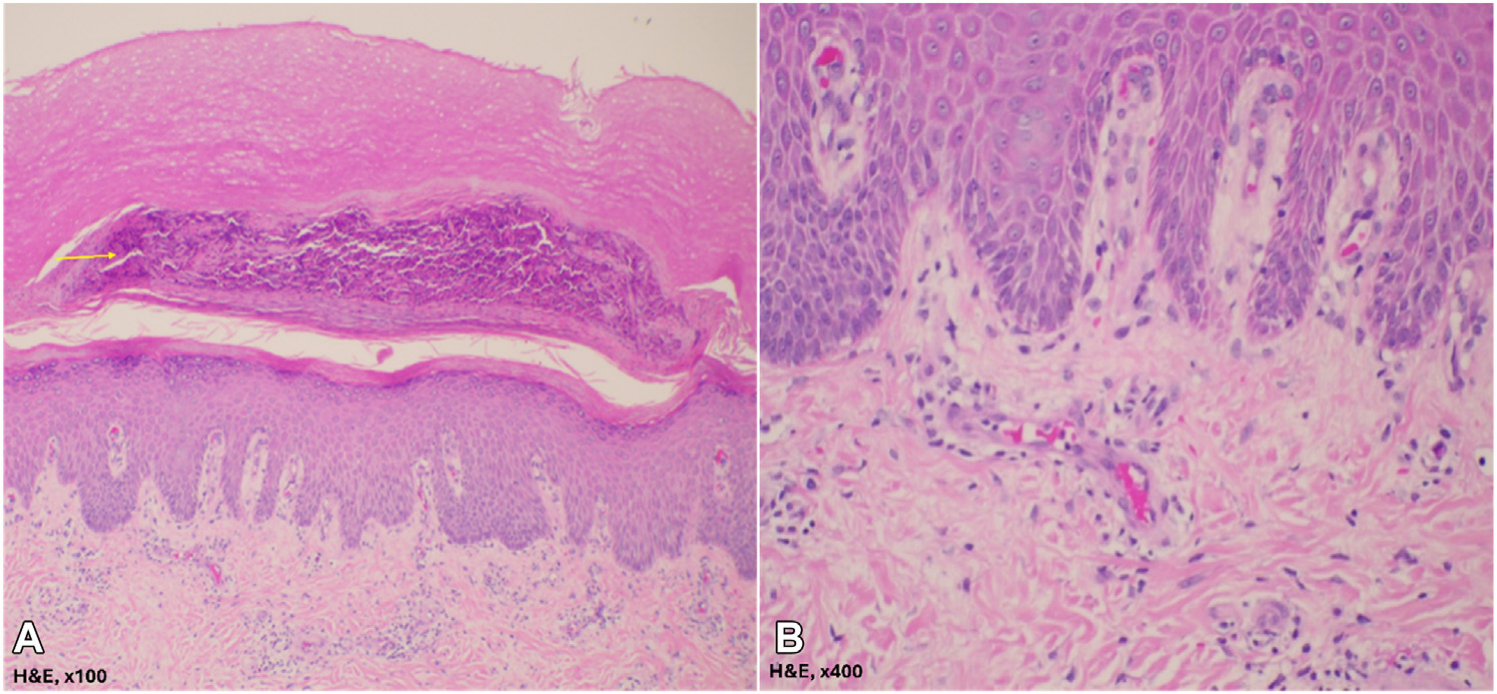
Skin biopsy (H&E staining) of the epidermis shows mounds of parakeratosis with neutrophils (*yellow arrow*) (**A**) and proliferation of blood vessels with superficial perivascular lymphocyte infiltration in the dermis (**B**).

The initial diagnostic workup, including a complete blood count, serum creatinine, and liver function tests, returned normal results. A potassium hydroxide preparation from skin scraping was negative for fungal infection. A punch biopsy from the foot revealed epidermal hyperplasia with parakeratosis containing neutrophils, consistent with psoriasis (Fig 2). Inflammatory markers showed elevated serum levels of IFN-α, MCP-1, IL-6, IL-10, IL-18, and IL-33, while TNF-α and IL-17A levels remained normal (Table I). These findings confirmed a diagnosis of paradoxical psoriasis attributed to TNF-α inhibitor therapy.

**Table I:** Initial presentation and 6-month follow-up of inflammatory markers and serum cytokine levels compared to normal ranges

Inflammatory marker	Normal range	At the first presentation	Six-month follow-up
ESR (mm/h)	0-20	33	24
CRP (mg/L)	0-5	6.89	2.48
Serum inflammatory cytokine (pg/mL)			
IL-1 beta	1.59-13.88	3.63	4.5
IFN-alpha	1.88-7.60	10.61	2.13
IFN-gamma	1.30-17.09	12.29	6.74
TNF-alpha	2.47-22.28	7.11	2.5
MCP-1	40.73-318.12	418.90	429.65
IL-6	2.19-12.88	19.85	3.89
IL-8	2.00-80.71	38.19	15.07
IL-10	1.96-28.48	39.13	2.84
IL-12p70	0.79-8.70	7.53	0.43
IL-17A	0.81-16.84	3.41	4.21
IL-18	50.78-166.58	277.10	298.96
IL-23	4.55-16.30	6.67	2.86
IL-33	9.65-104.64	279.70	52.42

*CRP,* C-reactive protein; *ESR*, erythrocyte sedimentation rate; *IFN*, interferon; *MCP-1*, monocyte chemoattractant protein-1.

**Fig 3:**
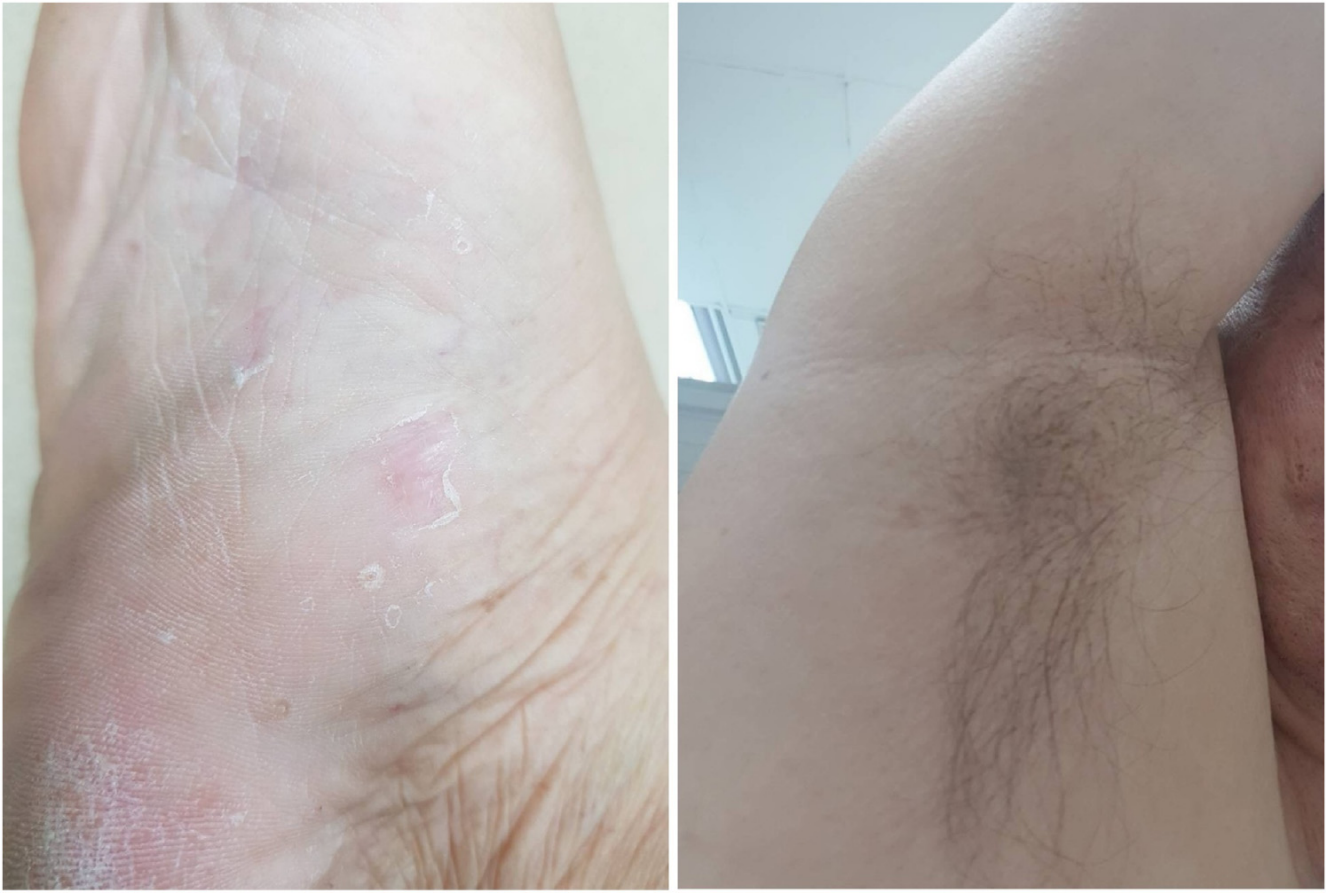
Rashes on the patient's sole (*left*) and axilla (*right*) completely resolved 1 month after discontinuing golimumab.

Upon diagnosis, golimumab was discontinued to prevent further exacerbation of the skin condition. The patient was prescribed a topical ointment containing 0.05% clobetasol propionate, leading to complete resolution of the rash within 1 month (Fig 3).

However, 3 months after discontinuing golimumab, the patient developed ankle arthritis and back stiffness not alleviated by nonsteroidal anti-inflammatory drugs. To address these symptoms, secukinumab, an IL-17A inhibitor effective against both psoriasis and ankylosing spondylitis, was initiated with a loading dose of 150 mg weekly for 4 weeks, followed by monthly maintenance doses. Regular follow-up visits at 2 and 6 months indicated that the patient remained in remission from ankylosing spondylitis and had no new skin lesions.

## Discussion

In previous literature, infliximab is the most frequently reported TNF-α inhibitor to cause psoriasiform eruptions (50% to 60%), followed by etanercept (10% to 30%), adalimumab (10% to 35%), certolizumab pegol (1%), and golimumab (0.5%). The overall prevalence is approximately 1.3% to 5%. The median onset time for these reactions is approximately 6-12 months.^4^ Our case is one of the few documenting paradoxical psoriasis resulting from golimumab.

This report presents a thorough serum cytokine analysis of a patient with TNF inhibitor-induced psoriasis, which contrasts with classic psoriasis characterized by the Th17 pathway and cytokines such as IL-17 and IL-23.^3^ Previous meta-analyses have shown significantly elevated serum levels of TNF-α, IFN-γ, IL-2, IL-6, IL-8, IL-18, and IL-22 in psoriasis patients, while levels of IL-1β, IL-4, IL-10, IL-12, IL-21, and IL-23 showed no significant differences.^5^ Some studies have reported increased serum IL-17 levels^6^; however, this finding was significant primarily in male cohorts according to the referenced meta-analysis.^5^

In our case, we observed elevated IFN-α levels instead of activation of the IL-17/23 axis, indicating a unique inflammatory pathway in TNF inhibitor-induced psoriasis. This phenomenon is linked to PDCs, which can produce excessive IFN-α when TNF-α regulation is disrupted. PDCs have been found in early psoriatic lesions and even in normal-looking skin before plaque formation, but are absent in healthy skin.^7^ Elevated IFN-α levels in the dermal tissues of patients treated with TNF antagonists suggest a significant role in skin dysregulation.^8^

Inhibiting TNF-α also appears to affect Th1 lymphocyte trafficking, increasing their presence in peripheral circulation.^3^ PDC-derived IFN-α aids in the maturation of myeloid dendritic cells and activates Th1 cells. This elevation of IFN-α, alongside Th1 cell migration, is likely to contribute to the paradoxical development of psoriasis during TNF antagonist therapy, necessitating further research to clarify these complex relationships.

Our cytokine analysis showed elevated levels of IL-6, IL-18, and IL-33, demonstrating a complex network of interactions in TNF inhibitor-induced psoriasis. The elevated levels of IL-6 and IL-18 also correlate with inflammatory responses, as observed in the control psoriasis group in the referenced meta-analysis.^5^ However, IL-33's role in psoriasis remains controversial; some studies suggest it may alleviate the condition,^9^ while others indicate it could exacerbate it.^10^ Normal TNF-α levels in our case, despite TNF inhibitor use, may relate to the timing of cytokine measurements, occurring 2 months postgolimumab.

To our knowledge, our case is among the few that have examined serum cytokines in paradoxical psoriasis resulting from anti-TNF therapy. These findings suggest that targeting IFN-α may offer new therapeutic options for this form of psoriasis; however, additional studies assessing cytokine profiles in these patient groups are essential to confirm this approach. While secukinumab effectively managed the patient's ankylosing spondylitis after discontinuing anti-TNF therapy, its effect on psoriasis remains unclear, especially without initial IL-17 elevation. Further research into cytokine interactions is important for developing tailored therapies for this unique form of psoriasis.

## Conflicts of interest

None disclosed.
